# Repair of *O*^6^-methylguanine adducts in human telomeric G-quadruplex DNA by *O*^6^-alkylguanine-DNA alkyltransferase

**DOI:** 10.1093/nar/gku659

**Published:** 2014-07-30

**Authors:** Lance M. Hellman, Tyler J. Spear, Colton J. Koontz, Manana Melikishvili, Michael G. Fried

**Affiliations:** Department of Molecular and Cellular Biochemistry, Center for Structural Biology, University of Kentucky, Lexington, KY 40536, USA

## Abstract

*O*^6^-alkylguanine-DNA alkyltransferase (AGT) is a single-cycle DNA repair enzyme that removes pro-mutagenic *O*^6^-alkylguanine adducts from DNA. Its functions with short single-stranded and duplex substrates have been characterized, but its ability to act on other DNA structures remains poorly understood. Here, we examine the functions of this enzyme on *O*^6^-methylguanine (6mG) adducts in the four-stranded structure of the human telomeric G-quadruplex. On a folded 22-nt G-quadruplex substrate, binding saturated at 2 AGT:DNA, significantly less than the ∼5 AGT:DNA found with linear single-stranded DNAs of similar length, and less than the value found with the telomere sequence under conditions that inhibit quadruplex formation (4 AGT:DNA). Despite these differences, AGT repaired 6mG adducts located within folded G-quadruplexes, at rates that were comparable to those found for a duplex DNA substrate under analogous conditions. Repair was kinetically biphasic with the amplitudes of rapid and slow phases dependent on the position of the adduct within the G-quadruplex: in general, adducts located in the top or bottom tetrads of a quadruplex stack exhibited more rapid-phase repair than did adducts located in the inner tetrad. This distinction may reflect differences in the conformational dynamics of 6mG residues in G-quadruplex DNAs.

## INTRODUCTION

Cellular DNA is exposed to many chemical and physical agents that can modify DNA bases and/or backbone structures. Human cells growing under ordinary laboratory conditions experience DNA damage at a rate of ∼20 000 lesions per day (1), making effective DNA repair essential for genomic integrity. One particular type of damage is the alkylation of *O*^6^-atoms in guanine residues. This modification is cytotoxic and mutagenic in cells and carcinogenic in model organisms (1–3). In human cells, *O*^6^-alkylguanines are repaired by the *O*^6^-alkylguanine DNA alkyltransferase (AGT, also known as methylguanine methyltransferase, MGMT) (4,5). Ironically, this enzyme also protects tumor cells against chemotherapeutic DNA-alkylating drugs (5–8). AGT inhibitors are currently in clinical trial with the aim of improving the efficacy of alkylating agents in cancer chemotherapy (9–11).

Human AGT is a small, globular, monomeric protein (*M*_r_ = 21 519), constitutively expressed in normal cells (5,12). It binds with similar affinities and cooperativities to linear single- and double-stranded DNAs (13). Binding is moderately cooperative on both substrates (13,14), but while cooperative binding is likely to be part of the lesion-search mechanism (15), it is not required for repair (5,16). AGT catalyzes reactions in which a single alkyl group is transferred from the *O*^6^-position of guanine, or less efficiently, the *O*^4^-position of thymine (4,17), to an active-site cysteine residue. This returns the DNA base to its unmodified state, but the alkylated enzyme is permanently inactivated by this reaction and rapidly degraded *in vivo* (18,19).

**Figure 1.**
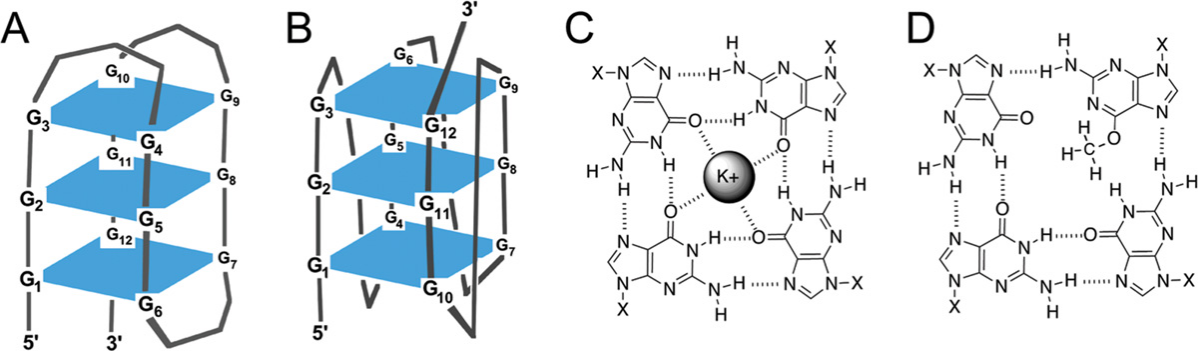
Schematic diagrams of G-quadruplex DNAs. (**A**) Backbone diagram of a three-quartet quadruplex in the antiparallel ‘basket’ conformation. Guanine residues are numbered; other residues are located at the vertices of the connecting line segments. The planes of the G-quartets are shown as rectangles in projection. (**B**) Backbone diagram of a three-quartet quadruplex in the all-parallel ‘propeller’ conformation. Drawing conventions as in (A). (**C**) Schematic representation of hydrogen bonding and ion binding within a single G-quartet. Here X represents the sugar-phosphate backbone. Redrawn from (23). (**D**) Schematic diagram showing potential effect of *O*^6^-methylation of a single guanine on hydrogen bonding and ion binding within a G-quartet. Here X represents the sugar-phosphate backbone.

Much less is known about AGT function with non-canonical DNA structures such as the G-quadruplexes that can form in DNA containing the human telomeric repeat sequence (TTAGGG)_x_ (20,21), and that protect chromosome ends and play roles in telomere maintenance (22). G-quadruplex structures can form if one guanine in a stack of quartets is methylated at the *O*^6^-position, although the methyl group interferes with hydrogen bonding within the quartet, and quite possibly also the binding of a counterion near the center of the affected quartet (Figure 1C, 23). The result is destabilization of the quadruplex, with respect to the corresponding unmethylated structure (24). Together these features suggest that there might be a role for AGT activity in the maintenance of the unmethylated form of the telomere sequence. On the other hand, differences in charge density, geometry and base-stacking energies that distinguish quadruplex from duplex structures led us to predict that AGT would be unable to bind and repair *O*^6^-methylguanines in G-quadruplex structures. The experiments described below tested these predictions.

## MATERIALS AND METHODS

### DNAs

Oligonucleotides (Table 1) were synthesized by The Midland Certified Reagent Company (Midland, TX, USA) and were supplied in high-performance liquid chromatography-purified form. Samples were dissolved in 10 mM Tris–HCl (pH 7.6), 1 mM ethylenediaminetetraacetic acid (EDTA) (TE buffer) and further purified by phenol extraction. Residual phenol was removed by ether extraction and residual ether was removed by evaporation. Samples were dialyzed overnight at 4°C against TE buffer, supplemented, where indicated, with 75 mM KCl, or 75 mM triethanolamine–HCl (TEA), and/or 5 mM MgCl_2_. In all preparations, the free base form of EDTA was used to avoid unintentional introduction of Na^+^ or K^+^. Buffers containing Na^+^ were not used in these studies because K^+^ is the dominant intracellular monovalent cation (25) and because preliminary sedimentation velocity experiments indicated that some G-quadruplex-forming sequences gave >1-fold when Na^+^ was the dominant buffer cation (Hellman, unpublished result). Before use, quadruplex-forming oligonucleotides were melted by heating to 95°C, slowly cooled to 37°C and then annealed overnight at 37°C. Sample concentrations were determined spectrophotometrically, using extinction coefficients supplied by the manufacturer. The fractions of DNAs active as substrates in repair reactions were determined by incubation for 8 h with excess AGT under K^+^-free (non-folding) conditions, followed by electrophoresis (see below). Measured in this way, DNAs used were >92% active (results not shown).

**Table 1.** Oligonucleotide sequences

Name	Sequence MW (Da)	Sequence (5’ to 3’)
22wt	7565.3	Fl-AGG GTT AGG GTT AGG GTT AGG G
22wtx	6966.8	AGG GTT AGG GTT AGG GTT AGG G
G1	7579.2	Fl-A**G**G GTT AGG GTT AGG GTT AGG G
G2	7579.2	Fl-AG**G** GTT AGG GTT AGG GTT AGG G
G3	7579.2	Fl-AGG **G**TT AGG GTT AGG GTT AGG G
G4	7579.2	Fl-AGG GTT A
		
		
		
		**G**G GTT AGG GTT AGG G
G5	7579.2	Fl-AGG GTT AG**G** GTT AGG GTT AGG G
G5x	6980.7	AGG GTT AG**G** GTT AGG GTT AGG G
G6	7579.2	Fl-AGG GTT AGG **G**TT AGG GTT AGG G
G11	7579.2	Fl-AGG GTT AGG GTT AGG GTT AG**G** G
25-mer G11	8500.9	Fl-AGG GTT AGG GTT AGG GTT AG**G**GTTA
26-mer	8532.7	Fl-AGT CAG TCA GTC AGT CAG TCA GTC AG
24-mer NarI	7326.8	GGG TCA TTT G**G**C GCC TTT CGA TCC
		123 456
24-mer NarI complement	7380.2	GGA TCG AAA GGC GCC AAA TGA CCC

Fl denotes 6-carboxyfluorescein labeled at 5’-end.

The boldface **G** denotes *O*^6^-methylguanine (6mG).

22wtx and G5x are unlabeled (fluorescein-free) version of the 22wt and G5 DNAs.

### AGT preparations

AGT and its catalytically-inactive C145A mutant were expressed and purified as previously described (26). Samples were dialyzed at 4°C against 10 mM Tris (pH 7.6), 100 mM KCl containing either 2 mM 2-mercapto-ethanol or 200 μM TCEP [Tris(2-carboxyethyl)phosphine hydrochloride] as indicated. Protein concentrations were determined spectrophotometrically, using *ϵ*_280_ = 3.93 × 10^−4^ M^−1^ cm^−1^ (27). The wild-type enzyme preparations used were >95% active in transfer of methyl groups from single-stranded *O*^6^-methyl guanine-labeled oligonucleotides (28). The C145S enzyme had no detectable alkyltransfer activity, although it was >95% active in DNA binding (15).

### Circular dichroism spectroscopy

Spectra were obtained at 10°C using a Jasco J-810 spectropolarimeter fitted with a thermostated sample holder. Scans were acquired at 20 nm s^−1^; at least four scans were averaged in each dataset. DNA concentrations were typically 3–5 μM. When spectra were taken with AGT, samples contained a 10-fold molar excess of AGT over DNA. Difference spectra for nucleic acids bound by AGT were obtained by subtracting the spectrum of free protein from the spectrum of protein/nucleic acid mixture. Thermal melting profiles were obtained with DNA samples annealed as described above and equilibrated at 0°C for 10 min prior to measurement. Temperature was increased at 1°C min^−1^ with data collected at 295 nm, every 0.5°C. Parallel temperature scans made at 0.5°C min^−1^ superimposed on those made at 1°C min^−1^, indicating that thermal and conformational equilibria had been attained (results not shown).

### Analytical ultracentrifugation

Sedimentation velocity and equilibrium measurements were made using an XL-A analytical ultracentrifuge (Beckman-Coulter) fitted with an An-60 rotor, operating at 4°C. Data fitting used numerical solutions of the Lamm equation implemented in the SEDFIT program (29,30). Experimental *s* values (*s*_T,B_) were converted to *s*_20,w_ values using Equation (1) (31).

(1) \begin{equation*} s_{{\rm 20,w}} = s_{{\rm T,B}} \frac{{\left( {1 - \overline \upsilon \rho _{{\rm 20,w}} } \right)}}{{\left( {1 - \overline \upsilon \rho _{{\rm T,B}} } \right)}}\frac{{\left( {\eta _{{\rm T,B}} } \right)}}{{\left( {\eta _{{\rm 20,w}} } \right)}} \end{equation*}

Here, $\overline \upsilon$ is the partial specific volume (0.54 ml g^−1^ for G-quadruplex DNA; 0.55 ml g^−1^ for single-stranded DNA) (32), $\rho$ is the buffer density (measured using a Mettler density meter) and $\eta$ is the buffer viscosity, calculated from tabulated values using the program SEDNTERP ((31), available from http://bitcwiki.sr.unh.edu/index.php/Main_Page). Viscosities of TEA solutions were estimated by interpolation of concentration- and temperature-dependent values tabulated by Maham *et al.* (33) to the concentration used in our experiments (75 mM), and then extrapolation of the temperature-dependent results to the experimental temperature (4°C).

Sedimentation equilibrium measurements were made over a range of rotor speeds (12 000–35 000 rpm), at 4°C. Equilibrium was held to have been attained when scans taken 6 h apart superimposed without net difference. In a system containing protein in molar excess over DNA, where [protein] >> *K*_d_ for DNA binding, the principal species will be free protein (P) and the saturated protein–DNA complex (P*_n_*D) with *n* protein molecules bound to each DNA. At sedimentation equilibrium, the radial absorbance distribution of this molecular system is given by Equation (2) (27).

(2) {\fontsize{8}{}{\fontsize{8}{11}\selectfont\begin{eqnarray*} A(r){=}\alpha _{\rm P} \exp \left[ {\sigma _{\rm P} \left( {r^2{-}r_0^2 } \right)} \right]{+}\alpha _{{\rm P}_n {\rm D}} \exp \left[ {\sigma _{{\rm P}_n {\rm D}} \left( {r^2{-}r_0^2 } \right)} \right]{+}\zeta \end{eqnarray*}}}

Here *A(r)* is the absorbance at radial position *r*, $\alpha _{\rm P}$ and $\alpha _{{\rm P}_n {\rm D}}$ are the absorbance components of protein and the protein–DNA complex at *r*_0_, the reduced molecular weights of free protein and protein–DNA complex are $\sigma _{\rm P} = M_{\rm P}(1- \overline {v}_{\rm P} \rho ) \omega ^{2} / (2RT)$ and $\sigma _{{\rm P}_{n}{\rm D}} = (nM_{\rm P} + M_{\rm D}) (1 - {\overline \nu}_{{\rm P}_{n}{\rm D}})\omega ^{2} / (2RT)$ where *M*_P_ and *M*_D_ are the molecular weights of protein and DNA, respectively, $\overline \nu$ is the partial specific volume, $\rho$ the solvent density, $\omega$ the angular velocity, *R* the gas constant, *T* the absolute temperature and $\zeta$ is a baseline offset. The partial specific volume for the protein–DNA complex was calculated using Equation (3).

(3) \begin{equation*} \overline \nu _{{\rm P}_n {\rm D}} = \frac{{\left( {nM_{\rm P} \overline \nu _{\rm P} + M_{\rm D} \overline \nu _{\rm D} } \right)}}{{\left( {nM_{\rm P} + M_{\rm D} } \right)}} \end{equation*}

### Repair kinetics

Reactions with telomere sequence DNAs were carried out at 4°C, in TE buffer, containing 75 mM KCl and 5 mM MgCl_2_ (for folded G-quadruplexes) or 75 mM TEA (for unfolded telomere DNAs). Reaction mixtures typically contained 4 μM AGT and 0.25 μM 6-carboxyfluorescein (FAM) -labeled DNA. Aliquots were removed at intervals and reactions quenched by phenol extraction (28). Residual phenol was removed by ether extraction and residual ether evaporated under vacuum. Where necessary, samples were adjusted to contain 20 mM KCl and 0.5 mM MgCl_2_. Reaction products were resolved by native gel electrophoresis at 4°C in 20% polyacrylamide gels containing 40 mM Tris–acetate (pH 7.6), 20 mM KCl and 0.5 mM MgCl_2_. Resolved species were detected with a Typhoon 9400 imager (Amersham Biosciences) and quantified using ImageQuant software (GE Healthcare).

Single-stranded 6mG-containing NarI-24-mer DNA (Table 1) was 5’-end labeled with ^32^P (34). Free [γ^32^P]-ATP was removed by dialysis and the labeled DNA was annealed with 1.1 equivalents of unlabeled complementary oligo (sequence given in Table 1). Repair reactions included 0.037 μM DNA and 0.074 μM AGT in buffer containing 40 mM Tris–acetate (pH 7.9 at 20°C), 100 mM potassium acetate, 2 mM magnesium acetate and 2 mM dithiothreitol (DTT) ; reactions were quenched at selected intervals with 0.2% sodium dodecyl sulphate (SDS). Samples were extracted with phenol and ether as described (28). Samples were adjusted to 10 mM magnesium acetate and digested with NarI endonuclease. Products were resolved on 20% polyacrylamide gels and radioactive bands were detected by phosphorimager. Band quantitation used the program ImageQuant as described above.

## RESULTS

### Quadruplex structures are compact and predominantly monomeric

**Figure 2.**
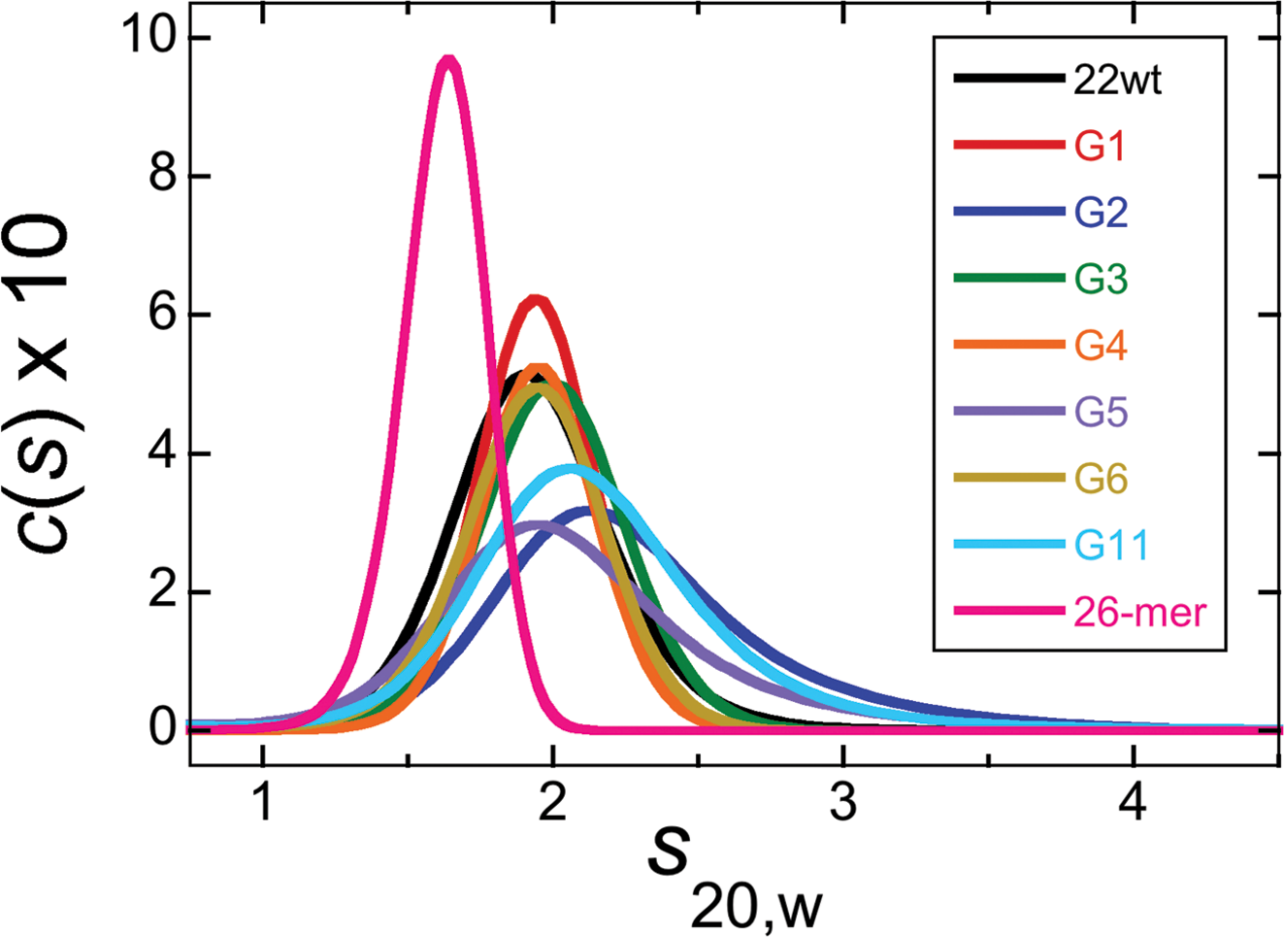
Sedimentation coefficient distributions for telomere sequence and single-stranded DNAs. Sedimentation velocity data were measured at 4°C and 40 000 rpm in 10 mM Tris–HCl (pH 8.0), 1 mM EDTA supplemented with 75 mM KCl and 5 mM MgCl_2_. DNA concentrations were ∼0.9 μM (*A*_260_ ∼0.25). Smooth curves are *c*(*S*) distributions calculated with the program Sedfit (30,35).

Sedimentation velocity experiments were performed to determine whether the telomere-sequence DNAs containing 6mG modifications formed compact structures. Dissolved in TE buffer containing 75 mM potassium chloride, both unmodified and 6mG variants of the telomere sequence sedimented as single species with *s*_20,w_ ∼2.0 (Figure 2A, Table 2). These values are similar to one previously reported for the quadruplex form of the unmodified 22-mer (22wt) (24). Molecular weight (*c*(*M*)) distributions (35) for the 22wt DNA and for sequences containing 6mG at positions G1, G3, G4 or G6 (outer quartet locations) returned mean values (7850 < *M*_r_ < 8760), slightly greater than the sequence molecular weights of the DNAs (*M*_r_ ∼ 7600), supporting the notion that these species were primarily monomeric (Tables 1 and 2). However, DNAs with 6mG modifications at inner-quartet positions (G2, G5 and G11) gave higher apparent molecular weights (9695 < *M*_r_ < 10 938) suggesting the presence of a modest mole-fraction of higher oligomeric species. The sedimentation coefficient (*c*(*S*)) distributions for these DNAs were also broader than those of the others (Figure 2), consistent with models in which monomers and multimers exchange rapidly during sedimentation.

**Table 2.** Sedimentation velocity data

	*s*_T,B_	*s*_20,w_^a^	*M*_r_ (Da)	*f*/*f*_0_^b^	Axial ratio
22wt^c^	1.47 ± 0.21	1.93 ± 0.27	8768 ± 1815	1.36 ± 0.17	2.1
G1^c^	1.49 ± 0.15	1.94 ± 0.20	7969 ± 1179	1.35 ± 0.12	2.0
G2^c^	1.72 ± 0.31	2.25 ± 0.41	10,938 ± 2924	1.17 ± 0.18	∼1
G3^c^	1.53 ± 0.19	2.00 ± 0.25	7850 ± 1445	1.32 ± 0.15	1.5
G4^c^	1.49 ± 0.15	1.95 ± 0.20	7853 ± 1198	1.35 ± 0.13	1.9
G5^c^	1.56 ± 0.28	2.03 ± 0.37	9695 ± 2568	1.29 ± 0.20	1.3
G6^c^	1.48 ± 0.19	1.93 ± 0.25	8036 ± 1489	1.36 ± 0.15	2.1
G11^c^	1.63 ± 0.28	2.13 ± 0.37	10,051 ± 2557	1.24 ± 0.18	1.3
26-mer^c^	1.08 ± 0.02	1.41 ± 0.15	8247 ± 1652	1.87 ± 0.05	8.6
G1^d^	0.62 ± 0.11	1.29 ± 0.23	7943 ± 1219	1.95 ± 0.29	14.3
G5^d^	0.65 ± 0.14	1.33 ± 0.32	7720 ± 1311	1.86 ± 0.32	12.6
22wtx^e^	0.79 ± 0.13	1.24 ± 0.20	6813 ± 1556	1.98 ± 0.28	10.1
22wtx^c^	1.50 ± 0.2	1.95 ± 0.26	6925 ± 1245	1.37 ± 0.18	2.1
G5x^e^	0.77 ± 0.09	1.20 ± 0.14	6541 ± 1064	2.05 ± 0.21	11.1
G5x^c^	1.54 ± 0.19	2.02 ± 0.25	7090 ± 1093	1.31 ± 0.12	1.4

^a^*s*_20,w_ values converted from *s*_T,b_ values. Ranges are 95% confidence intervals (31).

^b^*f/f_0_*, buffer densities and viscosities were calculated using Sednterp (31), with $\bar v$ = 0.54 ml g^−1^ for G-quadruplex DNAs (32) and $\bar v$ = 0.55 ml g^−1^ for the 26-mer ssDNA (36).

^c^In 10 mM Tris–HCl (pH 8.0), 1 mM EDTA and 75 mM KCl.

^d^In 10 mM Tris–HCl (pH 8.0), 1 mM EDTA and 75 mM TEA.

^e^In 10 mM Tris–HCl (pH 8.0) and 1 mM EDTA (acid form).

Telomere-sequence 22-mers sedimented more rapidly (*s*_20,w_ ∼2.0) than a 26-nt single-stranded DNA (*s*_20,w_ ∼1.4), suggesting that they were more compactly-folded than the higher molecular weight linear molecule. We used SEDFIT to calculate the frictional ratios (*f/f*_0_) of our DNAs; telomere DNAs measured in TE + 75 mM KCl buffer had frictional ratios in the range 1.17 ≤ *f/f*_0_ ≤ 1.37; this contrasted with the value for the single stranded 26-mer in the same buffer (*f/f_0_* ∼1.9; Table 2). The Perrin relation, implemented in SEDNTERP, was used to estimate the axial ratios (*a*/*b*) of hydrodynamically-equivalent prolate ellipsoids. This returned ratios of 1 ≤ *a*/*b* ≤ 2.1 for telomere DNAs in K^+^-containing buffer and 8.6 for linear 26-mer DNA. While these axial ratios rely on an assumed ensemble-average geometry, they provide a useful idea of the extent of compaction that telomere DNA undergoes as it folds.

### K^+^-dependent secondary structure formation

Potassium ions have been shown to stabilize the quadruplex fold in other telomere-sequence DNAs (37), so it was of interest to determine whether they played such a role with our sequences. Sedimentation of G1 and G5 DNAs in TE buffer containing 75 mM TEA–HCl returned frictional ratios 1.95 and 1.86, respectively, for which ellipsoid hydrodynamic models predict axial ratios of 14.5 and 12.6, respectively. Comparable results were obtained with 22wtx and G5x sequences in K^+^-free TE buffer (Table 2), supporting the notion that these telomere DNAs are substantially unfolded in the absence of K^+^.

**Figure 3.**
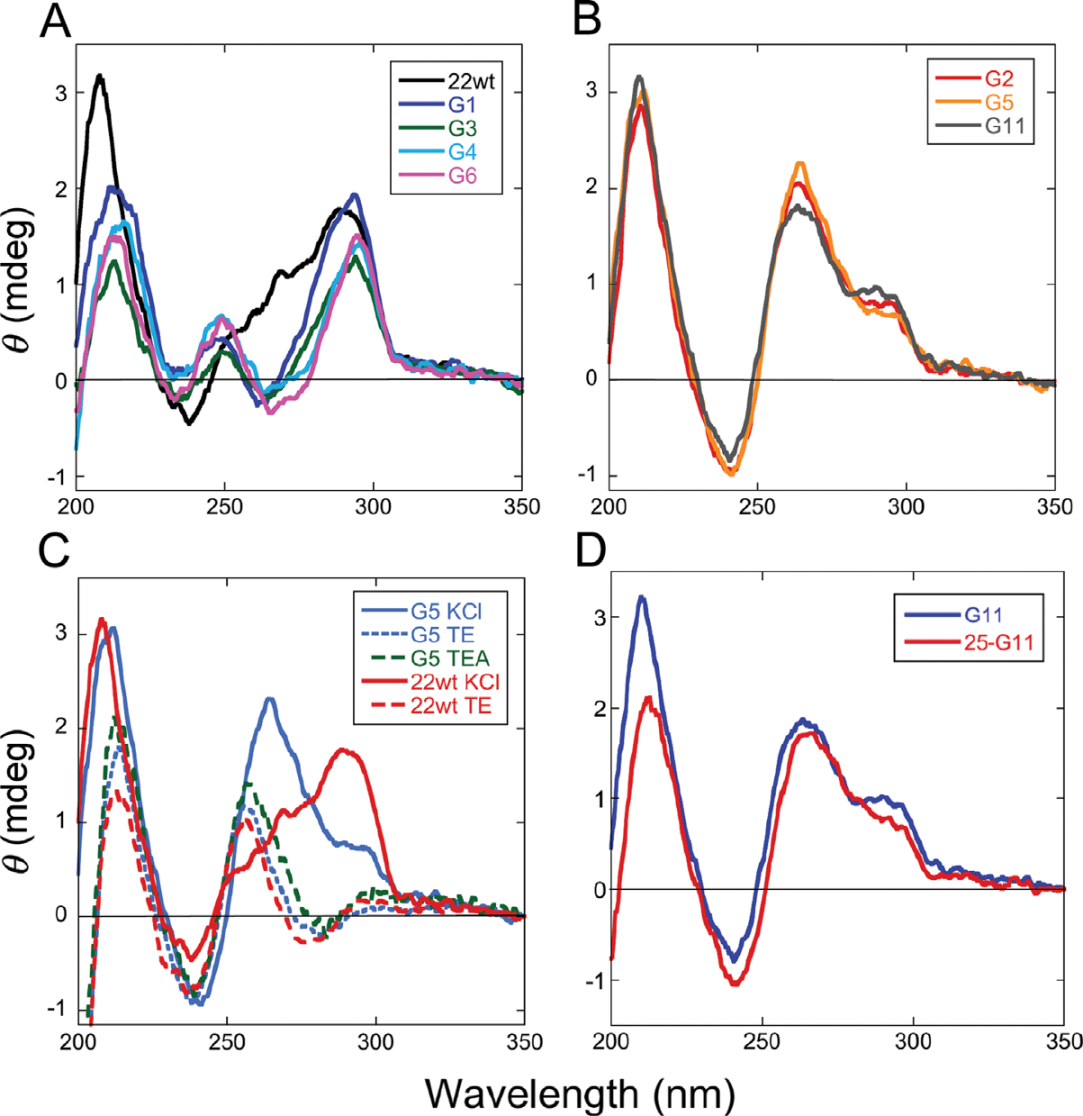
Circular dichroism spectra of telomere-sequence DNAs. (**A**) The 22wt and telomere sequences containing 6mG at outer tetrad positions G1 or G3 or G4 or G6, as indicated in the legend. Spectra were taken at 10°C in buffer containing 10 mM Tris–HCl (pH 8.0), 1 mM EDTA, 75 mM KCl and 5 mM MgCl_2_. (**B**) Spectra of telomere sequences containing 6mG at inner tetrad positions G2, G5 or G11, as indicated in the legend. Solution conditions were the same as in (A). (**C**) Spectra of 22wt and G5 sequences taken at 10°C in 10 mM Tris–HCl (pH 8.0), 1 mM EDTA (TE buffer) either alone or supplemented with 75 mM KCl or with 75 mM TEA, as indicated in the legend. (**D**) Comparison of spectra for the 22-mer G11 and 25-mer G11 DNAs. Spectra were taken at 10°C in 10 mM Tris–HCl (pH 8.0), 1 mM EDTA (TE buffer) and 75 mM KCl. DNA concentrations were 5 μM in all measurements.

Circular dichroism (CD) spectroscopy was used to further characterize the folding of these DNAs. In TE + 75 mM KCl buffer, the unmodified 22wt DNA and sequences with 6mG at residues G1, G3, G4 and G6 had CD maxima near 295 nm and minima near 235 nm (Figure 3A). These features correspond well to spectra previously observed for antiparallel strand quadruplex DNA (38). They also resemble previously-determined spectra of a folded form of the human sequence (24). In contrast, spectra for telomere sequences with 6mG at positions G2, G5 and G11 have distinct maxima near 260 nm (Figure 3B); maxima in this wavelength range have been described for parallel G-quadruplex DNAs (38,39). Finally, the CD spectra of 22wtx and G5x DNAs in K^+^-free buffers differ significantly from those obtained in the presence of K^+^, indicating the presence of conformations that are distinct from the quadruplex fold (Figure 3C). Spectra obtained in TE buffer and TE buffer containing 75mM TEA–HCl were similar, indicating that the corresponding conformations are not unique to low [salt] conditions.

**Figure 4.**
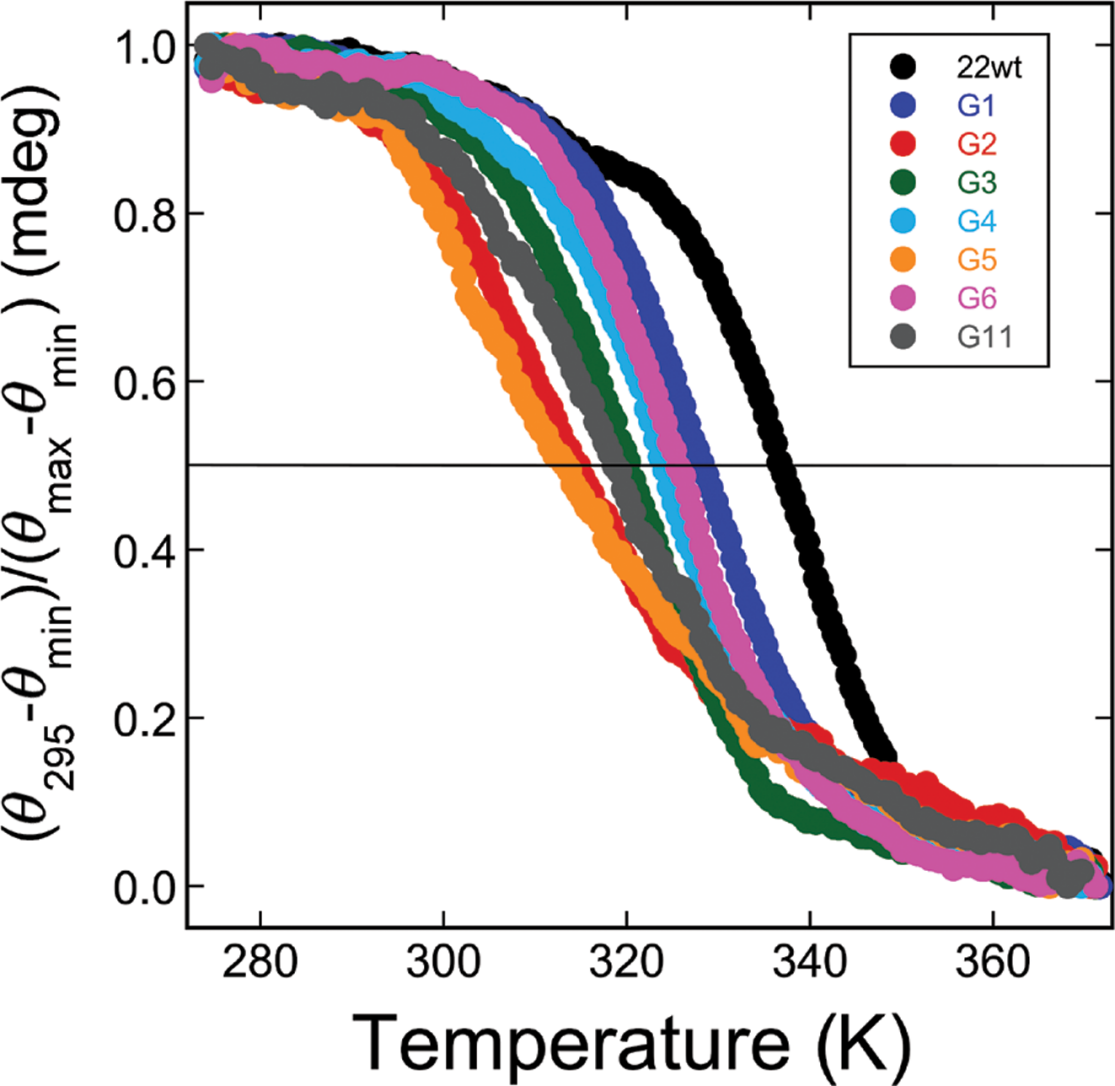
Thermal melting of G-quadruplex DNAs detected by circular dichroism. Samples were annealed at 37°C in 10 mM Tris–HCl (pH 7.6), 1 mM EDTA, 75 mM KCl, cooled slowly to 20°C for handling and then equilibrated at 0°C for 10 min prior to the start of analysis. Temperature was increased at 1°C min^−1^ with dichroism amplitudes measured at 295 nm, every 0.5°C.

Thermal denaturation profiles were obtained by monitoring CD changes at 295 nm (Figure 4). Under these buffer conditions, all DNAs had well-defined thermal transitions with low-temperature baselines that extended to ∼293°K, allowing sample handling at just-below room temperature without risk of denaturation. Transition midpoint (*T*_m_) temperatures ranged from 337°K (22wt DNA) to 313°K (G5 DNA), in the order 22wt > G1 > G6 > G4 > G3 > G11 > G2 > G5. Thus, folded forms of 6mG-containing DNAs had lower thermal stabilities than that of the unmodified DNA, and folded DNAs with 6mG residues in outer tetrads (G1, G3, G4, G6) were more stable than those with 6mG residues in inner tetrads (G2, G5, G11). This trend is consistent with and extends earlier observations made with telomere-sequence DNAs containing 6mG at positions G1, G2 and G3 (24). Together these hydrodynamic, spectroscopic and melting results are consistent with models in which these DNAs undergo K^+^-dependent folding to attain compact quadruplex conformations.

### AGT binds G-quadruplex structures

**Figure 5.**
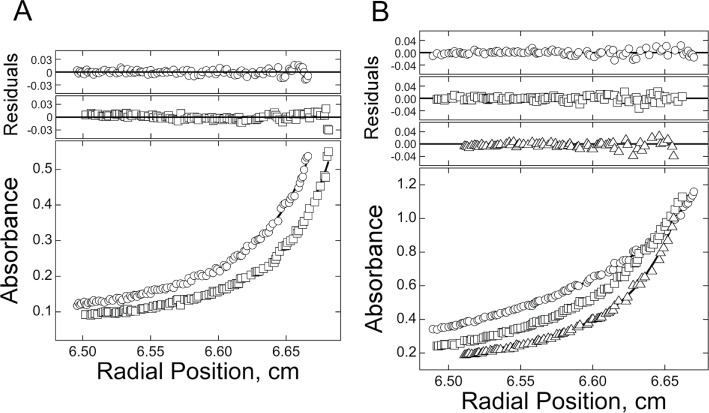
Sedimentation equilibrium analyses of AGT binding to telomere-sequence DNAs. (**A**) Radial concentration distributions for G5 DNA (0.8 μM) plus C145A AGT (12 μM), measured at 4°C and 25 000 (open circles) or 35 000 rpm (open squares), in buffer containing 10 mM Tris–HCl (pH 8.0), 1 mM EDTA, 75 mM KCl and 5 mM MgCl_2_. (**B**) Radial concentration distributions for G5 DNA (0.8 μM) plus C145A AGT (12 μM), measured at 4°C and 12 000 (open circles), 16 000 (open squares) or 20 000 rpm (open triangles), in buffer containing 10 mM Tris–HCl (pH 8.0) and 1 mM EDTA. The smooth curves represent global fits of Equation (2) to datasets obtained under each set of buffer conditions; the small, randomly distributed residuals (upper panels) indicate that the model embodied in this equation accounts well for the molecular weight distributions of the samples.

To discover whether K^+^-dependent DNA folding affects AGT interaction, binding densities were evaluated at sedimentation equilibrium under conditions of protein excess (Figure 5). Parallel experiments were conducted in TE buffer containing 75 mM KCl and in K^+^-free buffer, using the unmodified sequence 22wtx and the 6mG-containing sequence G5x (Table 1). Because wild-type AGT rapidly converts *O*^6^-methylguanine to unmodified guanine (3,15), the catalytically-inactive C145A mutant AGT was used for these experiments. This protein binds unmodified linear DNAs with stoichiometries and affinities that are indistinguishable from those of the wild-type enzyme (15,27). Data were fit using Equation (2), which embodies a model of binding saturation in which the dominant species are free protein and a protein–DNA complex. High-quality fits with small, randomly-distributed residuals show that this model is consistent with the data. Global analyses of datasets obtained at 3 rotor speeds and two dilutions of each protein–DNA mixture returned stoichiometry values of 1.96 ± 0.14 AGT/22wtx DNA and 1.82 ± 0.21 AGT/G5x DNA, in K^+^-containing buffer. Parallel analyses carried out in K^+^-free buffer returned values of 4.12 ± 0.27 AGT/22wtx DNA and 4.38 ± 0.16 AGT/G5x DNA in good agreement with results obtained with single-stranded DNAs of similar length but different sequence (13). Together, these findings suggest that DNA folding to form the G-quadruplex reduces the number of binding sites available to AGT.

### Retention of the quadruplex fold in the presence of AGT

**Figure 6.**
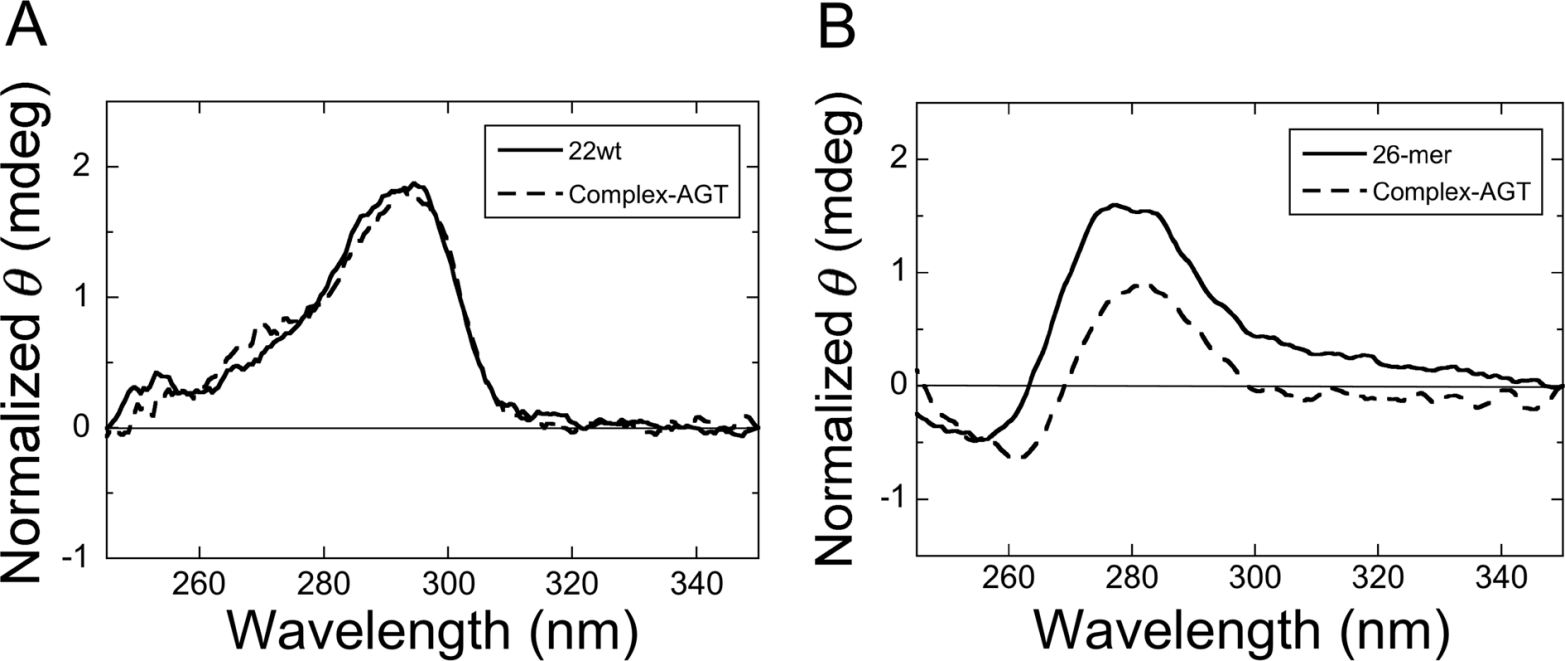
Circular dichroism spectra and difference spectra of AGT–DNA mixtures. (**A**) Spectrum of 22wt DNA (solid line) and difference spectrum (spectrum of AGT-22wt DNA mixture minus that of AGT alone; dashed line). The 22wt DNA and C145A-AGT concentrations were 5 and 50 μM, respectively, in buffer consisting of 10 mM Tris–HCl (pH 8.0), 1 mM EDTA, 75 mM KCl and 5 mM MgCl_2_. (**B**) Spectrum of the single-stranded 26-mer alone (solid line) and difference spectrum (spectrum of AGT-26-mer DNA mixture minus that of free AGT; dashed line). The 26-mer DNA and C145A-AGT concentrations were 3 and 30 μM, respectively. All spectra were recorded at 4°C.

We used CD spectroscopy to test whether AGT binding alters the secondary structures of our quadruplex DNAs. Difference spectra (CD_DNA+AGT_ – CD_AGT_) were obtained for the 22wt quadruplex in TE buffer containing 75 mM KCl and compared to spectra obtained at the same [DNA], but in the absence of AGT. As shown in Figure 6A, inclusion of AGT in the solution does not significantly change the DNA spectrum. This contrasts with the effect of AGT addition to a solution of the single-stranded 26-nt DNA, which results in a significant CD difference throughout the near-ultraviolet range (Figure 6B). These outcomes are simply explained by models in which AGT binding causes a substantial conformational shift in single-stranded 26-mer DNA, but not in the 22wt G-quadruplex.

### Repair of 6mG adducts in G-quadruplexs

**Figure 7.**
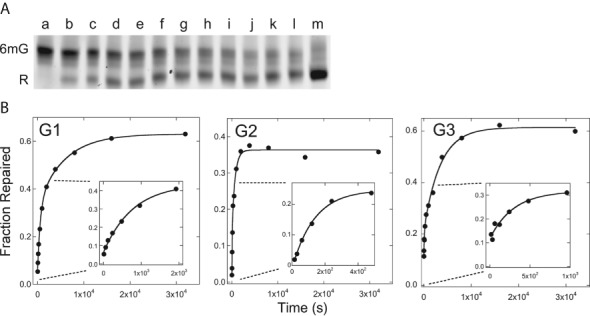
Time course of alkyltransfer in G-quadruplex DNAs. (**A**) Reaction substrate and product detected by electrophoresis. AGT (4 μM) was mixed with G1 DNA (0.25 μM) to start the reaction and samples were removed at intervals. Reactions were quenched by phenol extraction and DNAs were resolved by electrophoresis in a 20% gel cast and run in 40 mM Tris–acetate (pH 7.6), 1 mM EDTA, 20 mM KCl and 0.5 mM MgCl_2_. Lane a contains unrepaired G1 DNA; lanes b–l show reaction products sampled at 30, 60, 300, 600, 1200, 1800, 2700, 3600, 5400, 7200 and 10 800s. Lane m contains the 6mG-free 22wt quadruplex. (**B**) Time courses of representative repair reactions. Panels are labeled with the identities of the DNAs tested. Mole fractions of reactant and product bands were determined by fluorimetry. The smooth curves are fits of the time evolution of product formation, using Equation (4). Insets show the initial phases of each reaction in enlarged detail.

We took advantage of the observation (24) that short quadruplex DNAs containing 6mG residues migrate more slowly in native gel electrophoresis than do homologous quadruplexes lacking 6mG. Shown in Figure 7A, wild-type AGT added to solutions containing 6mG-quadruplex DNA gradually converts the low mobility (methylated) form into one with a higher mobility indistinguishable from that of the non-methylated 22wt DNA. In contrast, alkyltransfer-inactive C145A AGT does not produce a detectable shift (not shown). We interpret the mobility change as evidence of DNA repair. Mole fractions of repaired and unrepaired DNAs were quantified by fluorimetry; representative reaction time courses are shown in Figure 7B. The smooth curves are fits using Equation (4), which embodies a two-phase kinetic model:

(4) \begin{equation*} {{F}} = {A}_1 (1 - \exp ( - {\rm k}_1 {t})) + {A}_2 (1 - \exp ( - {\rm k}_2 {t}))\end{equation*}

Here, *F* is the mole fraction repaired, *A*_1_ the amplitude of the fast phase, (mole fraction scale rate constant k_1_) and *A*_2_ the amplitude of the slow phase (mole-fraction scale rate constant k_2_). The correspondence of fitted curves with data shows that this model accounts well for the observed time courses. For assays carried out on telomere DNAs that had been annealed in the presence of K^+^, single-phase models gave large systematic deviations from the data, while models with three phases gave adequate fits, but returned amplitudes for the third phase (*A*_3_) equal, within-error, to zero (result not shown). Thus, a two-phase mechanism appears to be the most parsimonious model consistent with the repair time course for folded telomere DNAs.

**Figure 8.**
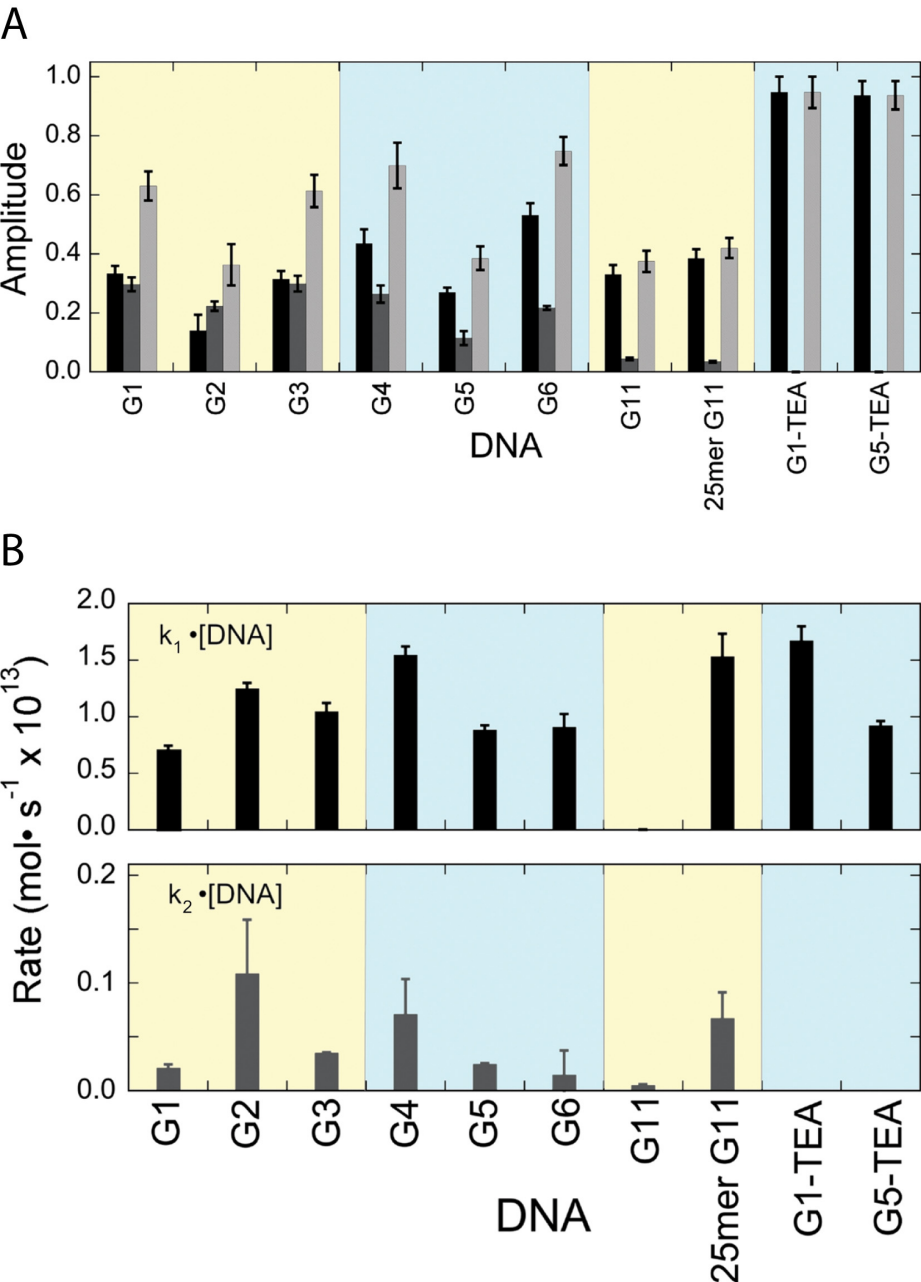
Summary of kinetics results for the repair of telomere-sequence DNAs by AGT. (**A**) Amplitudes of kinetic phases. Fast phases (represented by *A*_1_ in Equation 4) are graphed as black bars. Amplitudes of slow phases (*A*_2_ in Equation 4) are graphed as medium gray bars. The sums of amplitudes (*A*_1_ + *A*_2_) are shown as light gray bars. No slow phases were detected for G1 and G5 DNAs in TEA buffer. (**B**) Reaction rates for the fast phase (upper panel) and the slow phase (lower panel). Fast-phase rates were similar for all DNAs except the 22-mer G11. Extending that sequence by 3 nt at the 3’-end (25-mer G11) gave a DNA that was repaired with a rate similar to those of 6mG residues at other positions. Fast-phase rates are similar for folded DNAs (KCl buffer) and unfolded DNAs (TEA buffer). Rates for slow phases are ∼1/10 to ∼1/60 of those of the corresponding fast phases. No slow phase was detectible for DNAs in TEA buffer.

For G-quadruplexes annealed and repaired in buffer containing KCl, a comparison of the amplitudes of the fast and slow reaction phases with the extent of repair is informative (Figure 8A). The amplitudes of the fast phase and reaction extent after long incubation (8.8 h) vary with position within the quadruplex, with greater values at positions G1, G3, G4 and G6 (located in outer tetrads, Figure 1), and smaller values for positions G2 and G5 (inner tetrads). Repair amplitudes for the inner tetrad residue G11, both in the standard 22-mer sequence and in an elongated 25-nt version, were similar to those at the other inner positions. This pattern suggests models in which the stacking of G-quartets influences the amplitude of the fast phase of the reaction as well as the overall extent of repair.

A contrasting kinetic pattern was seen with G-quadruplex DNAs annealed and incubated with AGT in a buffer containing triethanolamine chloride (TEA) in place of KCl. For both G1 (outer tetrad) and G5 (inner tetrad) DNAs, the slow kinetic phases were suppressed and extents of repair were significantly greater (∼95% in TEA- but ∼63% in K^+^ buffer for G1, and ∼94% in TEA but ∼39% in K^+^ buffer for G5; Figure 8A). Taken with evidence that G-quadruplexes are substantially unfolded in the absence of K^+^ (Table 2), these results suggest that the fast phase of repair may correspond to a fraction of 6mG residues that is immediately available for interaction with AGT, while the slow phase may correspond to residues that become available as a result of a conformational shift in DNA or protein.

With one exception, the rates of the fast phase of G-quadruplex repair in K^+^-containing buffer are quite uniform. Under our reaction conditions, the range of repair rates was 0.7 × 10^−13^ mol·s^−1^ ≤ k_1_[DNA] ≤ 1.7 × 10^−13^ mol·s^−1^ (Figure 8B, top), thus falling in the same range as the rates for G1 and G5 DNAs in K^+^-free TEA buffer. As G1 and G5 DNAs are at least partially unfolded in TEA buffer (Table 2), these results support the notion that the fast phase corresponds to repair of 6mG residues that are highly-available to AGT. The sole exception is residue G11, which was repaired at a much slower rate (k_1_[DNA] ∼ 6 × 10^−16^ mol·s^−1^). Residue G11 is the last-but-one at the 3’-end of the 22-nt sequence, and previous work has shown that residues near the 3’-ends of single-stranded and duplex DNAs are repaired more slowly than internal residues (40,41). To determine whether proximity to the 3’-end influenced repair rate, we tested a longer G11 DNA (25 nt including a 3’ extension, Table 1). Although CD spectra indicated that this DNA had a fold similar to that of the 22-mer G11 (Figure 3D), it was repaired much more rapidly, with a rate similar to those of other DNAs in our sample set (Figure 8B, top). We interpret this as evidence that proximity to the 3’-end slows repair in G-quadruplexes in much the same way that it does in linear DNAs.

Reaction rates for the slow kinetic phase of G-quadruplex repair span the range 0.015 × 10^−13^ mol·s^−1^ ≤ k_2_[DNA] ≤ 0.11 × 10^−13^ mol·s^−1^, Figure 8B, bottom. This is 11–60-fold slower than the fast phases for the corresponding DNAs. The rate of the slow phase of G11 repair is increased by a 3-nt extension (compare 22-mer and 25-mer rates), suggesting that the 3’-end effect operates in the slow phase of quadruplex repair as well as in the fast. Reaction amplitudes for the slow phase follow a pattern similar to that found for the fast phase, with greater values for outer tetrad residues than for inner tetrad residues (Figure 8A). Our current hypothesis is that this reflects the relative stacking free energies of residues in each tetrad, with the stacked conformations of inner tetrad residues (stacked both ‘above and below’) more stable than those of outer tetrad that engage in tetrad stacking on one side only.

### Repair of a 6mG adduct in a double-stranded DNA

**Figure 9.**
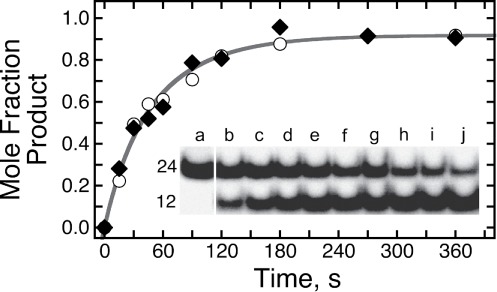
Time course of duplex DNA repair detected by NarI sensitivity. Inset. The 6mG-containing 24-mer duplex [0.037 μM, in 40 mM Tris–acetate (pH 7.9 at 20°C), 100 mM potassium acetate, 2 mM magnesium acetate, 2 mM DTT] mixed with AGT (final concentration 0.074 μM). Aliquots were withdrawn and quenched in 0.2% SDS after 15, 30, 45, 60, 120, 180, 270 and 360s, (samples b–i, respectively); unrepaired DNA is shown in sample a. DNA samples were deproteinized by phenol and ether extractions (28) and then digested with NarI endonuclease. Products were resolved by electrophoresis on a 20% gel. The singly-^32^P-labeled substrate duplex contains 24 bp and the ^32^P-labeled digestion fragment 12 bp; band designations give the lengths of the corresponding DNAs. Graph. Time profiles for two repair reactions carried out as described above. The smooth curve is a fit of Equation (4) to the combined data.

For comparison, we measured repair kinetics for a 6mG-containing 24-mer duplex (oligo sequences given in Table 1), using a NarI cleavage-susceptibility assay (28). This approach was necessary because we could not easily resolve the 6mG-carrying double-stranded DNA from that containing normal guanine, using standard electrophoresis methods (result not shown). NarI endonuclease is inactive against duplex DNA containing a 6mG residue at position 2 in its cognate sequence (42), but repair by AGT restores quantitative NarI cleavage. Shown in Figure 9 are gel data for the time-dependent repair of the 6mG-NarI 24-mer; the graph gives time course data for the experiment shown and for a second, parallel trial. The smooth curve is a fit of Equation (4) to the combined data, showing that a two-phase model is consistent with the kinetics of duplex DNA repair.

The rates observed for fast and slow phases of duplex DNA repair were k_1_·[DNA] = 0.31 ± 0.11 × 10^−13^ mol·s^−1^ and k_2_·[DNA] = 0.23 ± 0.10 × 10^−14^ mol·s^−1^, respectively. These are comparable to ones previously reported for duplex repair detected by the NarI assay, under similar conditions (15). They are also within the range that we found for repair of G-quadruplex DNAs (see above). However, important caveats apply to a comparison of these duplex and quadruplex repair rates, and these are discussed below.

## DISCUSSION

AGT binds and repairs *O*^6^-alkylguanine lesions in single-stranded and duplex DNAs with little difference in affinity or alkyltransfer rate (13,15). These modest differences, for substrates that differ in secondary structure, charge density, local stiffness and base-stacking stabilities (43) led us to ask whether AGT could function on substrates with other secondary structures. We chose the human telomeric sequence because it plays an important role in genome stability, is guanine-rich (50% G) when compared to the genomic average (∼21% G (44)) and because *O*^6^-methylation of residue G1, G2 or G3 had been shown to destabilize the folded quadruplex structure in that sequence (24). Our initial prediction, based on differences between the structures of duplex and quadruplex DNAs, was that repair of *O*^6^-methylguanine residues would be slowed or even halted by the quadruplex fold. The experiments described here were designed to test that prediction.

The major result of these studies is that AGT was able to repair 6mG residues in the context of G-quadruplex structures, and did so at generally similar rates for residues located at positions G1–G6 (numbered 5’ to 3’). On 22-nt DNA, the 6mG residue at position G11 was also repaired, though more slowly. Experiments with G11 DNA that contained a 3-nt extension at the 3’-end (25-mer G11, Table 1) led us to conclude that this slow rate reflected proximity to the 3’-end and was not a characteristic of position G11 in the quadruplex. Thus, all positions tested were susceptible to repair *in situ*, in a process that leaves the G-quadruplex structure intact and does not require base-excision or DNA repair synthesis. This work also revealed a number of intriguing consequences of substituting residues in the telomere sequence with 6mG and on mechanisms of AGT activity on quadruplex substrates. These are discussed briefly, below.

In our standard 75 mM KCl buffer, the unmodified telomere sequence and those with 6mG at positions G1–G6 and G11 sedimented as compact conformational ensembles (Table 2) and had CD spectra resembling ones previously found for G-quadruplexes (Figure 3). The CD spectra changed dramatically when K^+^ was deleted from the buffer or when it was substituted by TEA; in addition, in buffers lacking K^+^, the DNAs sedimented more slowly, consistent more extended structures. This dependence on K^+^ for folding is a well-documented feature of quadruplex structures. These results indicated that quadruplex folds were strongly represented in samples equilibrated in KCl buffer. The CD spectra of the 22wt DNA and DNAs with 6mG located outer tetrad positions (G1, G3, G4 and G6) were similar to that predicted for the antiparallel strand orientation (38,45). However, CD spectra for DNAs with 6mG at inner tetrad positions (G2, G5 and G11) were similar to that predicted for the parallel strand orientation (38,45). These differences suggest that the location of a 6mG residue can influence the relative stabilities of competing quadruplex folds, a notion supported by the melting data in Figure 4. Differences in quadruplex folds may influence enzyme interactions (see, for instance (46,47)) and thus functions available to quadruplexes in the cell.

The alkyltransfer-inactive C145S AGT bound both unmodified 22wt and G5-methyl G-quadruplexes (Figure 5), but with stoichiometries (∼2/DNA) that were significantly less than those found for single-stranded and duplex DNAs of similar length (typically 4–5 on single-stranded and duplex DNAs of similar length (13)). Circular dichroism difference spectra were consistent with models in which AGT binding had little effect on the conformation of the quadruplex (Figure 6). Together these data indicate that AGT can bind G-quadruplex-containing structures, but that its stoichiometry is limited by the quadruplex fold. The data did not specify the location of AGT-binding sites on quadruplex DNA; however, based on its ability to bind single-stranded DNAs (13), we suspect that the loops connecting quadruplex segments may provide sites of interaction. On the other hand, the ability to repair 6mG lesions within the quadruplex stack suggests that it also binds that structure, at least transiently. Future work will better define the sites of AGT interaction with quadruplex DNAs.

The kinetics of quadruplex repair were biphasic, with a fast phase similar to that found for unfolded telomere DNAs under analogous solution conditions, and a slow phase with rates 1/10 to 1/60 that of the fast (Figure 8). Our working hypothesis is that the fast phase reflects residues that are highly available and that the slow phase reflects the equilibration of residues between unavailable and available states. Reaction amplitudes for both fast and slow phases were greater for outer tetrad residues (G1, G3, G4, G6) than for inner residues (G2, G5, G11). This pattern would be expected if the kinetic stability of conformations in which the 6mG is unavailable (protected) depended on base-stacking interactions. As described above, the *O*^6^ methyl group has the potential to disrupt the hydrogen-bonding pattern of the G-quartet and also its ability to coordinate a potassium ion (Figure 1). Both effects are expected to reduce the equilibrium stability of the quadruplex fold and possibly to enhance the rate of exchange between stacked (unavailable) and unstacked (available) conformations of the 6mG. Because the methyl group is relatively small, we predict greater destabilization of quadruplex folds and greater rates of AGT repair will be found when DNAs containing *O*^6^-ethylguanine or larger substituents are tested.

Although the rates of the fast phases of G-quadruplex and duplex repair reactions are similar, this fact should be interpreted with caution. The quadruplex refolding and NarI assays operate under different reaction conditions and with different DNA sequences. However, similar binding affinities have been found for DNAs of different sequence and base composition, and modest changes in buffer composition appear to have limited effect on AGT affinity (12,14,27). Thus, we expect that the relatively small differences in buffer composition that distinguish these assays will exert only modest effects on observed repair rates. On the other hand, if the initial bimolecular binding steps are rate-limiting, repair rates should vary with [protein] and [DNA]. In this case, since the experiments with duplex and quadruplex DNAs were carried out at different [AGT] and [DNA], the similar repair rates that we have observed may be coincidental.

Our working hypothesis falls between these extremes. Both duplex and quadruplex kinetics are biphasic, consistent with models in which the initial binding step is not always rate-limiting. Mechanisms that can give two-phase kinetics include (i) ones in which the protein binds at either the lesion site (giving rapid repair) or a distal site (from which transfer is slow compared to repair); and (ii) ones in which transfer is not rate-limiting, but isomerization of protein or DNA between rapidly- and slowly-repaired states determines the distribution of rates. The model described above, in which base-stacking in G-quadruplexes limits access of AGT to the 6mG residue, is an example of (ii). If the rate-limiting step is unimolecular, then the rates of quadruplex and duplex repair will be independent of AGT and DNA concentrations. One such unimolecular reaction is a ‘sliding’ transfer between sites on a DNA molecule, like that observed with the excision-repair protein hOGG1 (48). A second is a conformational change in the DNA, like that proposed above. If a unimolecular step is rate-limiting, the rates we found with duplex and quadruplex DNAs will provide a useful comparison of the relative activities of AGT on those substrates. A more detailed characterization of AGT's reaction kinetics is needed to distinguish between these alternatives, and that work remains an important challenge for the future.

*In situ* antibody probing has provided evidence for the presence of quadruplex folds at telomeres *in vivo*, in both model organisms and human cells (49–51). Parallel *in vitro* work has shown that telomere structural proteins TEBPβ in *Oxytrichia* and Rap1 in *Saccharomyces cerevisiae* promote formation of quadruplex structures (52,53), while human POT1 protein destabilizes quadruplex structures and stimulates telomerase activity (22). More recently, several helicases have been discovered that may help to alleviate the problems that quadruplex-forming sequences may cause for DNA replication and RNA transcription (46,54–55). These and other results (reviewed in (21)) make a strong case for the presence and function of quadruplex structures *in vivo*, although details of the pathways in which they participate and the mechanisms of their functions remain to be discovered. Even less is known about how G-quadruplex-forming sequences are maintained, exposed as they are to many sources of chemical and physical damage *in vivo*, but progress along this line has started. Recent reports describe the activities of *Escherichia coli* MutS mismatch repair protein (56) and mammalian NEIL DNA glycosylases (57) on quadruplex substrates.

In this paper, we have added a characterization of AGT activities on quadruplex DNAs to the growing story, but much remains to be discovered. For instance, we do not yet know the relative affinities of AGT for quadruplex and duplex DNAs, nor how many binding sites of each type are available *in vivo*. Thus, we cannot predict how AGT will partition between telomere and non-telomere regions during its surveillance of the genome. Similarly, we do not know how susceptible G-rich telomere sequences are to modification by DNA-alkylating agents. The combination of high susceptibility to modification and slow repair might have significant consequences on telomere stability and functions. Filling these gaps in our knowledge will advance our understanding of alkylating-agent chemotherapy and also of telomere biology.
